# Understanding Older Adults’ Usage of Community Green Spaces in Taipei, Taiwan

**DOI:** 10.3390/ijerph110201444

**Published:** 2014-01-27

**Authors:** Eryn Pleson, Laura M. Nieuwendyk, Karen K. Lee, Anuradha Chaddah, Candace I. J. Nykiforuk, Donald Schopflocher

**Affiliations:** 1 Centre for Health Promotion Studies, School of Public Health, University of Alberta, 3-300 Edmonton Clinic Health Academy, Avenue 11405-87, Edmonton, AL T6G 1C9, Canada; E-Mails: pleson@ualberta.ca (E.P.); laura.nieuwendyk@ualberta.ca (L.M.N.); kkhlee2000@hotmail.com (K.K.L.); achaddah@rocketmail.com (A.C.); candace.nykiforuk@ulaberta.ca (C.I.J.N.); 2 Pratt Institute, New York, NY 10011, USA

**Keywords:** older adults, green spaces, parks, seniors, interviews, System for Observing Play and Recreation in Communities (SOPARC), age-friendly, physical activity

## Abstract

As the world’s population ages, there is an increasing need for community environments to support physical activity and social connections for older adults. This exploratory study sought to better understand older adults’ usage and perceptions of community green spaces in Taipei, Taiwan, through direct observations of seven green spaces and nineteen structured interviews. Descriptive statistics from observations using the System for Observing Play and Recreation in Communities (SOPARC) confirm that older adults use Taipei’s parks extensively. Our analyses of interviews support the following recommendations for age-friendly active living initiatives for older adults: make green spaces accessible to older adults; organize a variety of structured activities that appeal to older adults particularly in the morning; equip green spaces for age-appropriate physical activity; and, promote the health advantages of green spaces to older adults.

## 1. Introduction

The proportion of the population age 60 and older is growing rapidly throughout the World [1,2,3,4]. Physical and social environments can positively or negatively influence older adults’ physical activity and social connections [5,6,7]. Gilroy [8] argues that, for older adults, environments may become more constricting due to changing social and economic roles and decreased mobility. On the other hand, age-friendly environments can facilitate ‘aging in place’ by allowing older adults to live independently in their communities without being forced to move to age-segregated environments [8]. Timmer and Seymour [9] suggest that the creation of age-friendly environments reflects principles of equity, dignity, accessibility, conviviality, participation and empowerment.

Open spaces in urban areas (green spaces) can promote social engagement, physical activity, relaxation and interaction with nature [10]. They are generally accessible to the majority of the public and have limited user costs and time restrictions on utilization [10,11,12,13,14,15,16]. Green spaces may be particularly beneficial for older adults as they can provide safe opportunities to be active and interact with other people, while stimulating the mind and senses [17]. Despite the potential benefits older adults may be less likely to visit green spaces when compared to other age groups [10,18,19,20,21,22]. This juxtaposition of potential benefit with underutilization presents a challenge for the optimal use of green spaces in communities and for the promotion of physical activity in older adults.

The current body of literature has mainly explored older adults’ use of green spaces in North America and Britain [10,18,19,20,21,22]. Previous informal observations by members of the research team suggested that Taipei, Taiwan has an intensive and positive culture surrounding older adults’ use of green spaces that is rooted in traditional and artistic practices. This exploratory study sought: (a) to confirm that older adults in Taipei do make extensive use of community green spaces; and (b) to understand the reasons for this potential increased usage in the hope of informing age-friendly initiatives to support active living and physical activity in green spaces for older adults throughout the world.

## 2. Experimental Section

### 2.1. Data Collection

#### 2.1.1. Direct Observation

Seven community green spaces were observed and documented using Mackenzie and Cohen’s [23] System for Observing Play and Recreation in Communities (SOPARC) coding form with minor modifications to ensure the tool was applicable to the setting of the current study. These included: adding additional activity codes to better correspond with the types of activities undertaken by the local population (e.g., ballroom dance and tai chi); and, adding ‘multigenerational’ to the conditions of target areas to capture multigenerational interaction and/or use of space. Since ethnic background was not relevant to the objectives of this study, it was not recorded.

Sampling of green spaces began by choosing among the 12 administrative districts in Taipei, Taiwan those most heavily populated by older adults. These were then divided into higher and lower socioeconomic areas. Next, larger public parks were located on maps. This process relied heavily upon local contacts with detailed knowledge of the city.

Seven green spaces were then randomly selected as the green spaces to observe. Each green space was observed on a single weekday in April 2011. One green space was also observed a second time on Saturday in order to compare weekday to weekend use in one park observed to have high use by older adults on a weekday. Two parks were observed in the morning, two were observed during the afternoon, two were observed on the same day in both morning and afternoon, and the final park was observed on the same day in both morning and afternoon and again on Saturday in both morning and afternoon. Morning times varied between 5:45 AM and 12:30 PM while afternoon times varied between 3:00 PM and 6:30 PM.

Two SOPARC observers divided each green space into specific target areas of varying size. These target areas were standard locations chosen for their likelihood to provide opportunities for park users to be physically active. These included both relatively less developed areas (e.g., open field spaces, walking paths), as well as developed areas (equipped playgrounds, areas equipped with outdoor recreation equipment, basketball courts, open air structures) [23]. A total of 35 separate locations were observed across the seven parks.

The observers scanned each target area to obtain information (gender, age group, activity level and type of activity) about all park users within the target area. A total of 54 SOPARC separate scans were conducted. Each scan was timed to last 5 min. Contextual characteristics of each target area were also recorded to determine whether the area was accessible, usable, equipped, supervised, dark, empty, whether it provided organized activity, and whether it had multigenerational use. Six of these scans in three separate parks were independently performed simultaneously by the two observers. There were no disagreements in coding between the observers for these scans.

#### 2.1.2. Structured Interviews

In addition to the SOPARC observations of green space use, 19 structured video interviews of older adults were conducted. Interview participants who appeared to be 60 years of age or older were chosen at random from three green spaces. Each interview took between five and ten min to complete. Interviews were conducted in a combination of English and Mandarin Chinese with the help of a translator and later transcribed by the translator into English.

These interviews included questions about what types of activities older adults participated in at the green spaces, why they participated in these activities, who they participated with, what they liked about the green spaces, and what might make the green spaces better. In the interviews green spaces were referred to as public parks; a more commonly used term in Taipei culture.

Ethics approval was obtained from the University of Alberta Health Research Ethics Board and locally from the Taipei City Government (Public Works Department).

### 2.2. Data Analysis

#### 2.2.1. System for Observing Play and Recreation in Communities (SOPARC) Analysis

SOPARC data were entered into Statistical Package for the Social Sciences (SPSS) 18.0 for analysis. All observation forms were entered by one research assistant and double-checked by a second research assistant. Descriptive statistics, frequencies, and cross-tabulations were calculated.

#### 2.2.2. Interview Analysis

The translated interviews were coded using Feldman and Oberlink’s [24] framework for an elder-friendly community as a guide. Although intended to guide community design in the United States it was selected as a framework for analysis for the Taipei context to help guide how green spaces in North America can better support the health and wellbeing of older adults. The framework consists of four broad categories.

1. ‘*Addresses basic needs*’ encourages communities to provide adequate housing, safety, food, and services for older adults.
2. ‘*Promotes social and civic engagement*’ emphasizes the importance of opportunities for older adults to strengthen and build social relationships, be involved in the community, engage in meaningful work, and have their voices heard.
3. ‘*Optimizes physical and mental health and wellbeing*’ recommends that communities support healthy practices by offering activities that promote health and wellbeing, and increase the availability of preventative, medical, and social services for older adults.
4. ‘*Maximizes independence for frail and disabled*’ advises that community resources be used to ensure that frail and disabled older adults can live independently in their community.

For the 19 interviews in this study, these categories guided thematic analysis related to how community green spaces support the health and wellbeing of an aging population. Subthemes such as housing, safety, social engagement, community involvement, and health-promoting activities were based on some of the essential elements of this framework; however, the analysis also left room for the emergence of inductive subthemes related to age, physical health, and mental wellbeing.

## 3. Results and Discussion

### 3.1. System for Observing Parks and Recreation Communities (SOPARC) Results

All but one of the green spaces were considered usable and all were accessible and suitable for multigenerational use. Each also had at least one area that was equipped and supervised. A total of 1,231 individuals were observed performing a variety of different activities including walking, ballroom dancing, chi gong (a form of meditation/exercise based on traditional understanding of “chi” or energy), and group exercise classes. Table 1 provides a summary of the demographic composition and activity level of these individuals. Overall, the majority (61.3%) of people observed were judged to be older adults, primarily female (55%), generally walking (36.5%) or engaged in vigorous activity (36.5%).

**Table 1:** Demographics of all participants observed.

Variable		n (%)
Gender		
	Female	685 (55.6%)
	Male	542 (44.0%)
	Incomplete	4 (0.3%)
Age Group		
	Child (0–12 years of age)	153 (12.4%)
	Teen (13–17 years of age)	40 (3.2%)
	Adult (18–59 years of age)	265 (21.5%)
	Older Adult (60+ years of age)	755 (61.3%)
	Incomplete	18 (1.5%)
Activity Level		
	Sedentary	169 (13.7%)
	Walking	449 (36.5%)
	Moderate	94 (7.6%)
	Vigorous	449 (36.5%)
	Incomplete	47 (3.8%)

Table 2 provides a summary of the gender and activity level of the 755 older adults observed. Again there were more females (58.9%) who were walking (40.3%) or engaged in vigorous activity (35.2%).

**Table 2:** Demographics of older adult (age 60+) participants observed.

Variable		n (%)
Gender		
	Female	445 (58.9%)
	Male	309 (40.9%)
	Incomplete	1 (0.1%)
Activity level		
	Sedentary	68 (9.0%)
	Walking	304 (40.3%)
	Vigorous	266 (35.2%)
	Moderate	77 (10.2%)
	Incomplete	40 (5.3%)

Table 3 provides a summary of the primary and secondary activities that older adults were engaged in. As indicated in Table 3, older adults used community green spaces primarily to walk (40.3%) and for vigorous activities (35.2%), such as dancing or chi gong. Only 9.0% of older adults were sedentary. In addition, there were more female older adults (58.9%) than male older adults (40.9%) participating in activities in the green spaces.

**Table 3:** Summary of primary and secondary activities of older adults (age 60+).

**Primary Activity**		**n (%)**
	Walking	282 (37.4%)
	Dance class (including: ballroom dance, aerobic dance class)	127 (16.8%)
	Group exercise class (other than dance or chi-based class)	138 (18.3%)
	Eastern style exercise/movement classes based on traditional beliefs about Chi energy (e.g., chi gong, tai chi, yuan chi) whether individually or in a group	78 (9.9%)
	Sitting/socializing	21 (2.3%)
	Other (e.g., badminton, lawn bowling, Frisbee, stretching)	16 (2.1%)
	Activity at children’s playground area	15 (2.0%)
	Missing	78 (10.3%)
	TOTAL	755
**Secondary Activity**		**n (%)**	
	Running/cycling	207 (27.4%)
	Walking	68 (9.0%)
	Sitting/socializing	32 (4.2%)
	Exercise machines	21 (2.8%)
	Not available	427 (56.6%)
	TOTAL	755

Table 4 shows the relationship between open space usage by age group and time of day. The relationship is statistically significant (χ^2^ = 230.3, df = 4, *p <* 0.001). Older adults are much more likely to be present in the green spaces in the morning, while other age groups are more likely to be present in the afternoon. Almost the entire difference in overall usage is due to the heavy usage by older adults in the morning. The patterns remain the same when the analysis is repeated using only the parks which were observed in both the morning and afternoon. Table 4 also provides the subdivision by gender. The most important finding is that for adults and older adults, the ratio of females to males using the green spaces is higher in the morning. This contrasts with the greater use in the afternoons by younger males compared to females.

**Table 4:** Green space usage by age group, gender, and time of day.

			Age Group					
			Child (f/m/?)	Teen (f/m/?)	Adult (f/m/?)	Older Adult (f/m/?)	Uncertain (f/m/?)	Total
Time of Day	Morning	Count	65 (30/35/0)	1 (0/1/0)	148 (100/47/1)	630 (384/245/1)	11 (7/4/0)	855
		Row%	7.6%	0.1%	17.3%	73.7%	1.3%	
	Afternoon	Count	88 (28/60/0)	39 (2/37/0)	117 (69/46/2)	125 (61/64/0)	7 (4/3/0)	376
		Row%	23.4%	10.4%	31.1%	33.2%	1.9%	
Total		Count	153	40	265	755	18	1,231
		Row%	12.4%	3.2%	21.5%	61.3%	1.5%	

f/m/?: provides breakdown into female, male, and unknown gender.

Table 5 shows the pattern of organized activities tabulated by age group and time of day. Within each age group there is a statistically significant relationship between organized *versus* unorganized activities and morning *vs.* afternoon (χ^2^ = 15.1, df = 1, *p <* 0.001, χ^2^ = 25.5, df = 1, *p <* 0.001, χ^2^ = 121.8, df = 1, *p <* 0.001 for the three age groups respectively). In each case, a larger proportion of green space users were engaged in organized activities during the morning than during the afternoon. The differential was most marked for older adults. Overall there were many more adults and older adults engaged in organized activities in the morning compared to the afternoon, and approximately equal numbers of children and youth engaged in organized activities at each time. Table 5 also provides the subdivision by gender. For adults and older adults, the ratio of females to males previously noted in the morning is associated overwhelmingly to organized activities.

**Table 5:** Organized and unstructured activity by age group, gender, and time of day.

			Organized?		Total
			No (f/m/?)	Yes (f/m/?)	
Child & Teen	Morning	Count	29 (11/18/0)	37 (19/18/0)	66
		Row%	43.9%	56.1%	
	Afternoon	Count	92 (28/64/0)	35 (2/33/0)	127
		Row%	72.4%	27.6%	
	Subtotal	Count	121	72	193
		Row%	62.7%	37.3%	
Adult	Morning	Count	80 (45/34/1)	68 (55/13/0)	148
		Row%	54.1%	45.9%	
	Afternoon	Count	97 (56/39/2)	20 (13/7/0)	117
		Row%	82.9%	17.1%	
	Subtotal	Count	177	88	265
		Row%	66.8%	33.2%	
Older adult	Morning	Count	275 (116/158/1)	355 (268/87/0)	630
		Row%	43.7%	56.3%	
	Afternoon	Count	122 (58/64/0)	3 (3/0/0)	125
		Row%	97.6%	2.4%	
	Subtotal	Count	397	358	755
		Row%	52.6%	47.4%	
Combined	Morning	Count	384	460	844
		Row%	45.5%	54.5%	
	Afternoon	Count	311	58	369
		Row%	84.3%	15.7%	
	Total	Count	695	518	1,213
		Row%	57.3%	42.7%	

f/m/?: provides breakdown into female, male, and unknown gender.

In the one green space observed on both a weekday and a Saturday, there was approximately four times the number of green space users observed on the weekend morning than on the weekday morning. While the relative proportions by age group were very similar, as were the relative proportion of organized to unorganized activities, there were many more total individuals engaged in organized activities on Saturday morning. However there were no apparent differences in the afternoons between number and pattern of use in the weekday compared to the weekend. At least at this open space, Saturday seems to be a special day.

Table 6 shows the level of activity for both organized and unorganized activities tabulated by age group. Perhaps the most important part of this table is the sub-table for moderate to vigorous activities. Here it is clear that for older adults, organized activities are responsible for the large majority of moderate to vigorous activities, while this is less true for adults and for children and teenagers (χ^2^ = 117.0, df = 2, *p <* 0.001). Table 6 also provides the subdivision by gender. For older adults, moderate to vigorous organized activities are primarily a female activity while unorganized walking is primarily a male activity. This suggests that older males may walk while their spouses participate in organized activities either by choice or because the available organized activities may not appeal.

**Table 6:** Physical activity level by organization level and age group.

Activity Level			Organized?		Total
			No (f/m/?)	Yes (f/m/?)	
Sedentary	Child & Teen	Count	14 (10/4/0)	26 (12/14/0)	40
		Row%	35.0%	65.0%	
	Adult	Count	32 (24/8/0)	28 (20/8/0)	60
		Row%	53.3%	46.7%	
	Older adult	Count	48 (35/13/0)	20 (14/6/0)	68
		Row%	70.6%	29.4%	
	Subtotal	Count	94	74	168
		Row%	56.0%	44.0%	
Walking	Child & Teen	Count	40 (16/24/0)	9 (6/3/0)	49
		Row%	81.6%	18.4%	
	Adult	Count	110 (66/41/3)	4 (3/1/0)	114
		Row%	96.5%	3.5%	
	Older adult	Count	287 (114/172/1)	17 (11/6/0)	304
		Row%	94.4%	5.6%	
	Subtotal	Count	437	30	467
		Row%	93.6%	6.4%	
Moderate to	Child & Teen	Count	64 (12/52/0)	37 (3/34/0)	101
Vigorous		Row%	63.4%	36.6%	
	Adult	Count	33 (11/22/0)	54 (44/10/0)	87
		Row%	37.9%	62.1%	
	Older adult	Count	40 (14/26/0)	303 (235/68/0)	343
		Row%	11.7%	88.3%	
	Subtotal	Count	137	394	531
		Row%	25.8%	74.2%	

f/m/?: provides breakdown into female, male, and unknown gender.

We also classified the 35 observed locations into four types: equipped areas (children’s playground, exercise equipment, basketball court), areas with structures (bandstand, gazebo), paths, and open areas. Only organized activities were observed in areas with structures, and only unstructured activities were observed at equipped areas and almost exclusively on paths. A mixture of organized and unstructured activities was observed in open areas, though 84.7% of individuals observed were engaged in organized activities and of these 66.7% were older adults.

Finally, informal observation suggested that the organized activities for adults and older adults were often led by volunteer instructors. This was confirmed in conversations with interview participants. Activity organizers reported that the Parks Department allowed Taipei citizens to apply for a permit to teach a class at public parks which took only a few days to obtain and which was free of charge. Some instructors also provided a place to collect donations from participants.

Overall these observations strongly support the informal observations that green spaces in Taipei are widely used by older adults engaged in a wide range of physical activities. Older adults in Taipei are more likely to use green spaces during the morning and take part in organized activities almost exclusively in the morning. Furthermore these activities generally provide moderate to vigorous physical activities for older adults, and take place in open areas or in available structures such as gazebos or bandstands. There is also a substantial amount of walking by older adults, primarily on provided paths.

### 3.2. Interview Results

Table 7 provides a summary of the responses to direct interview questions. As indicated in Table 7, and consistent with the SOPARC observations, the most common activity in the green spaces interview participants reported was walking. Most of the 19 interview participants came to the green spaces every day and stayed one to two hours. Seven participants reported having visited these green spaces for over 10 years, and four of these indicated that they had been coming for over 20 years.

**Table 7:** Summary of direct responses to interview questions (*n =* 19).

**What types of activities do you like to participate in public parks? ^1^**		
	Walking	9
	Stretching	4
	Morning exercise	3
	Dancing	3
	Using equipment	3
	Chi gong	2
	Warm up	2
	Other (e.g., biking, petanque, badminton, frisbee)	8
**How often do you participate in these activities?**		
	Every day	9
	Almost every day	2
	5 to 6 times a week	4
	2 to 4 times a week	3
	Only when the weather is good	1
	Missing	1
**How long do you participate in these activities?**		
	Less than 1 h	4
	1 h	5
	1.5 to 2.5 h	6
	3 to 4 h	1
	Missing	3
**How long have you been coming to the public parks?**		
	6 months	1
	2 to 4 years	2
	More than 10 years	3
	20 to 25 years	2
	30 or more years	2
	Missing	9
**How do you get to the public parks?**		
	Walk	12
	Bike	3
	Motorcycle	2
	Missing	2
**How long did it take you to get to the public park?**		
	6 min or less to walk	4
	10 to 15 min to walk	5
	Over 15 min to walk	1
	3 to 5 min to bike	1
	10 to 15 min to bike	1
	1 km	1
	Missing	6
**What form of transportation do you use for regular daily activities? ^1^**		
	Walk	4
	Bike	2
	Bus or metro	4
	Motorcycle	1
	Missing	12
**Do you bring children or grandchildren with you to the public parks?**		
	Yes	7
	No	7
	Missing	5
**Do you have friends that exercise?**		
	Yes	8
	Some	1
	No	1
	Missing	9

^1^ Participants provided multiple responses to this question.

Table 8 presents a summary of the themes extracted from the open-ended responses of older adult park users. This summary of themes and example quotes is meant to provide an overview of participant responses which are explored in more detail in the discussion section.

**Table 8:** Summary of thematic analysis of interview responses.

Theme/Subtheme	Description	Example Quote(s)
**Addresses basic needs**		
Housing proximity	Two interview participants commented on the proximity of community green spaces as an influential factor in deciding where to live in Taipei. Participants conveyed that the green spaces were the reason why they ‘moved’ or ‘bought a house’ in the area.	“*I bought the house because it’s close to the park. People like to come here and exercise, that’s why we live close to the park.*”
	Three participants noted that living near the green spaces had some bearing on their decision to visit the spaces. One participant mentioned that living far away influenced why people chose not to visit.	“*This park is near my home.*” “*My neighbors think it’s too far to walk to the park, so they don’t come here.*”
Safety	Safety was also mentioned as a basic need that factored into participants’ decision to come to the green spaces. Three participants believed the green spaces were safe.	“*And this park is safe.*”
**Promotes social and civic engagement**		
Social engagement	Thirteen interview participants spoke of the social interactions that occur at the community green spaces.	“*I feel that our dancing group is like a big family, we love being here.*” “*Also, I like the friends I made here in the park.*”
	Two participants listed ‘socializing’ or ‘chatting with friends’ as one of the activities that they like to do while at these spaces.	“*I like to walk around the park, and I spend time with friends.*”
	Social engagement was part of the participants’ daily routine at the community green spaces and was described by one participant as ‘a habit’.	“*We like to come here to meet friends and exercise. It keeps us healthy, and it has become a habit to meet friends in the park.*”
Community involvement	Thirteen participants highlighted a wide range of community activities offered by the green spaces (e.g., praise dance, chi gong, petanque (a form of bowls resembling bocce), badminton, frisbee, yoga).	“*There are so many things happening here, for example, you can see people singing Taiwanese opera, children playing on the machines, older adults doing morning exercises…*”
	Responses indicated that these activities support community involvement after retirement. Three participants mentioned that they have a lot more spare time now compared to when they were in the work force.	“*I don’t have to work and I have a lot of time, so I wake up early and come to the park.*”
	Two participants contrasted their use of the green spaces against people they knew who ‘stayed at home all the time’ or are ‘couch potatoes’.	“*We are getting old and we have a lot of free time, we shouldn’t stay at home all the time.*”
	For three participants, the presence of group activities in the green spaces attracted new people to visit the spaces and join in the activities.	“*When I came to this park and saw this dance group, I found them interesting so I joined them.*”
**Optimizes physical and mental health and wellbeing**		
Age and physical health	Fourteen participants responded that they exercise to be healthy.	“*It’s good for your health, and you don’t get sick easily.*”
	Five participants spoke of the connection between age, exercise and physical health. This included the importance of physical activity to maintain health as you age.	“*I started exercise because I was getting old. I wanted to keep healthy.*”
	Two participants commented on how being active and ‘moving’ was what it meant to live.	“*Because we should move to live. We should exercise.*”
	One participant noted that physical activity helped older adults feel younger.	“*Because we are getting old, exercise makes us feel healthy and younger.*”
	Four participants expressed that they had more time to be physically active because of their age and the fact that they are retired.	“*And after I retired 5 years ago, I started to exercise. Before retirement, I was too busy at work; I didn’t have time to exercise.*”
**Optimizes physical and mental health and wellbeing**		
Health-promoting activities	Ten interview participants commented on how community green spaces support activities that promote health and wellbeing such as walking, bicycling, chi gong, yoga, dance, badminton and stretching.	“*Before the park [was] built I went mountain hiking every morning. And now I benefit from this park, I come here for the fresh air, and it’s a great place for people to do exercise.*”
	Participants also spoke to the green spaces offering structured activities as well as being a place participants can engage in unstructured activities of their own. Ten participants mentioned structured activities such as badminton, petanque, dance class, and yoga. Eleven participants mentioned unstructured activities such as jogging, walking, and stretching.	“*I came here and saw people doing Yoga, so I just joined them. I think Yoga is the best exercise for me.*”*;* “*I swing my arms for about an hour in the morning, and I come here to walk around the park for about 30 min… I don’t really have a schedule of doing exercise.*”
	Four participants commented on how the building and maintenance of the green spaces by the government specifically helped to support health-promoting activities.	“*The government has built many nice parks for us.*”
Mental wellbeing	In addition to the physical benefits, three participants also noted how engaging in park activities has had a positive effect on their mental wellbeing. Participants associated ‘happiness’ and ‘self-achievement’ with exercise.	“*Playing petanque makes me healthy and happy. Also, I feel self-achieved when I play petanque.*”
**Maximizes independence for frail and disabled**		
	Three interview participants conveyed how the activities and resources of the green spaces accommodate any disabilities and/or impairments that have accompanied old age.	“*But after going mountain hiking for 5 years, my knees got weaker, so I now don’t go mountain hiking. I walk around in this neighborhood instead.*”; “*I do all kinds of stretching [on] the equipment. We are getting old, and sometimes we have backache. Doing stretching will [relieve] the backache.*”
	Three participants acknowledged that park activities are good alternatives to the activities they use to enjoy when they were younger.	“*When I was young, I did all kinds of exercises. But after I am retired, I found petanque is an interesting and healthy exercise, also, it’s a good exercise for older adults.*”

### 3.3. Discussion

Our purpose was to understand older adults’ use of, and perceptions about, community green spaces in Taipei, Taiwan. The results from the SOPARC analysis confirmed earlier informal observations by research team members that many older adults used green spaces in Taipei and those older adults were the primary users of the green spaces in Taipei. The SOPARC observations provided the context for 19 structured interviews with older adult park users. The themes and sub-themes that emerged from interview analysis are discussed in greater detail below.

#### 3.3.1. Addresses Basic Needs

Previous research has indicated that older adults are more likely than younger people to remain in the neighborhood in which they have lived [25]. As a result, older adults rely on their neighborhood to support their needs [8,26]. For some participants in this study, the decision of where to live in Taipei was influenced by the proximity of community green spaces. In addition, proximity of housing to older adults residences’ influenced participants’ decision to actually utilize the green spaces, and living farther away was highlighted by participants as a reason why friends did not use the spaces. Being within walking distance positively influences older adults’ use of parks [27,28,29,30,31]. The majority of interview participants reported walking to the green spaces and indicated that it took 15 min or less to get to the space.

#### 3.3.2. Promotes Social and Civic Engagement

Kweon and colleagues [25] found that green spaces are significantly and positively associated with social integration among older adults by drawing older adults out of their homes and increasing the likelihood of informal face-to-face interaction. This social integration can lead to more neighborly activities and better relationships with friends and neighbors, which in turn strengthens older adults’ sense of community [25]. SOPARC data indicated that socializing is both a primary and a secondary activity for older adults in Taipei. Interview participants reported that social engagement was one of the main reasons that they came to the green spaces and was just as important as participating in physical activity.

**Figure 1:**
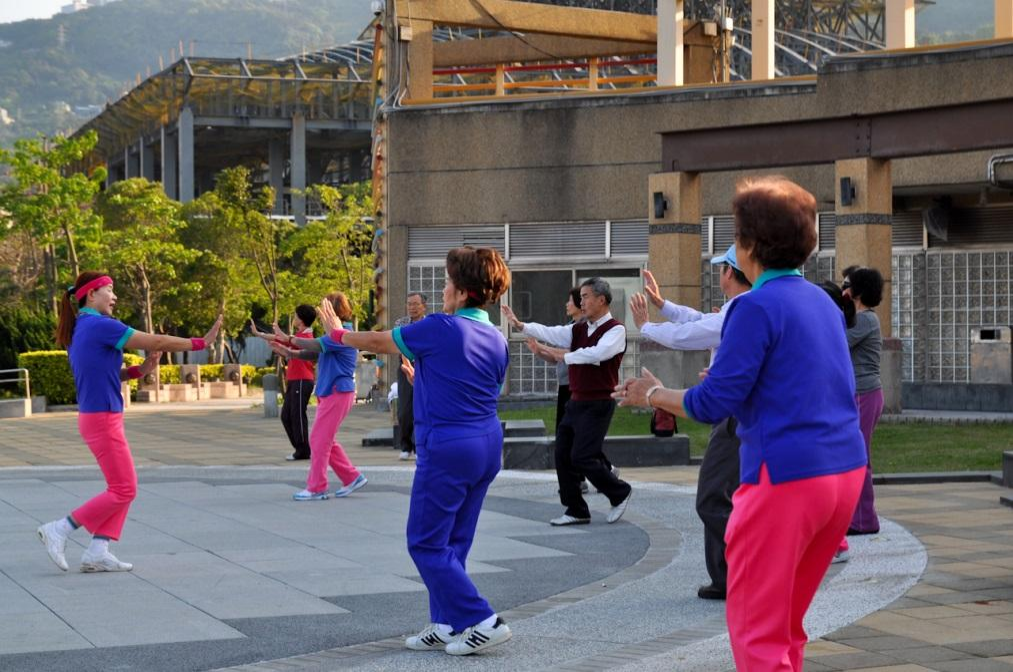
Example of Tai Chi class in a Taipei green space.

Despite having more free time [27], older adults often find themselves disconnected from their community due to changing economic and social roles [8]. For interview participants, local green spaces offered a way of filling free time with meaningful activities such as morning exercise, dance, chi gong, badminton, petanque and Frisbee. The SOPARC data showed that dance classes, group exercise and chi gong were among the primary activities of older adults (see Figure 1 for an illustrative example). It is evident from the interviews that these activities attracted many older adults to these green spaces. Individuals who have friends and family who are active are more likely to engage in physical activity [15,32]. Many participants in this study reported that their friends were also active and that this helped to encourage and support their participation in physical activity.

**Figure 2:**
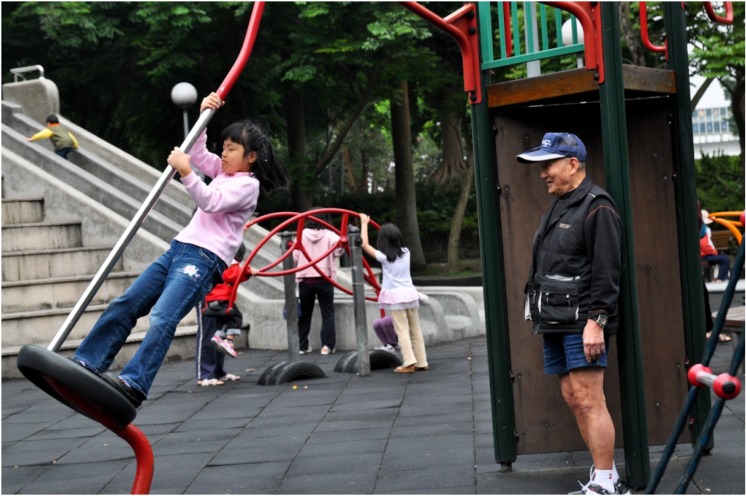
Example of intergenerational interaction in Taipei green space.

The caregiver role of grandparents influences older adults’ recreation preferences [21]. Seven interview participants reported bringing their grandchildren to the green spaces (see Figure 2 for an illustrative example). In Taipei, grandparents are often caregivers to grandchildren and the community green spaces provide opportunities for multigenerational interaction that support the cultural practices for childcare.

#### 3.3.3. Optimizes Physical and Mental Health and Wellbeing

Older adults who are active are able to maintain their functional ability and independence longer than those who are inactive [33]. In addition, physical activity is associated with improved quality of life and reduced risk for many chronic conditions such as cardiovascular disease, type 2 diabetes, osteoporosis, and numerous cancers [34,35,36]. The majority of older adults observed in the green spaces in this study were walking or engaging in moderate to vigorous exercise. Interview participants reported engaging in physical activity in order to remain healthy as they aged.

**Figure 3:**
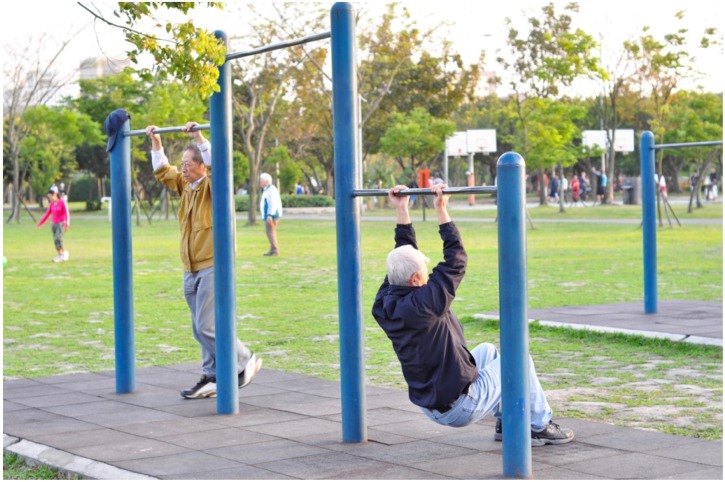
Example of older adults performing strength training on equipment in green spaces.

Parks that provide equipment and organized activities help to promote physical activity [37]. Community green spaces in Taipei offered a wide range of activities. In addition to structured activities, green spaces in Taipei also provide park users with the opportunity to engage in unstructured activities such as walking, bicycling, Frisbee and stretching and strength training on the equipment (see Figure 3 for an illustrative example).

Physical activity can also promote mental wellbeing. For example, physical activity has been found to have a protective effect against depression in older adults [38]. Interview participants frequently made the connection between participating in park activities and their mental health. Length of park stay can also have a positive effect on mental health, for example by reducing stress [39]. Over half the participants in the current study stayed at the green spaces for over an hour and went to the green spaces every day or nearly every day.

Green spaces themselves can also be associated with mental health benefits by improving moods [10,15] and reducing anxiety and sadness [10,40]. In this study, self-achievement was mentioned as a benefit to participating in activities at the green spaces. Engaging in both physical and non-physical activities at the green spaces may decrease the phenomenon that Hung and Crompton [27] describe as ‘rolelessness’ by bringing fulfillment to the daily lives of older adults.

#### 3.3.4. Maximizes Independence for Frail and Disabled

For many older adults, the aging process results in frailty and disability. By reducing barriers to being active, park activities can improve the functional capacity of frail and disabled older adults [6,41,42,43]. To accommodate these individuals, the green spaces in Taipei offer rehabilitation machines for light-resistance exercise and reflexology paths to improve balance.

Since individual preferences for recreation tend not to change over time [21], the protective effect of physical activity enables older adults to continue to engage in the activities they enjoy or modified versions of these activities. Participants in the current study described park activities in Taipei’s green spaces such as petanque and walking as good alternatives to the activities they used to participate in when they were younger and more able.

### 3.4. Lessons from Taipei

These results lead naturally to the following recommendations:

(1) *Make green spaces accessible to older adults*. In established communities ensuring that green spaces are in close proximity to older adult residences may not be easy to arrange, but supports can be put in place to ensure that these green spaces are accessible (e.g., providing transportation to these spaces or places close to these spaces). Ensuring that green spaces are available within walking distance to residential areas for older adults should become a standard part of city planning. Although a short walking distance to parks facilitates the use of these spaces for some older adults, further research may be needed to determine the effect walking distance has on park use for older adults with different physical capacities (e.g., use of a walker or wheelchair). In addition, future research should explore what distance and travel time older adults perceive to be too far to walk to access not only green spaces but also other amenities.
(2) *Provide opportunities for a variety of structured activities that appeal to older adults, particularly in the morning*. Practitioners should be encouraged to provide a variety of structured and unstructured activities in community green spaces to support older adult participation and community engagement. As in Taipei, activity instructors might be permitted to teach informal classes. While providing these types of activities may not be feasible where weather is a barrier to outdoor activities, there is the potential for these activities to be provided in indoor community park spaces (e.g., recreation facilities, malls, libraries) in the winter months. If such indoor spaces are located in close proximity to green spaces, all the better.
(3) *Equip green spaces for age-appropriate physical activity*. In this study, the SOPARC data revealed that green spaces were accessible, equipped and supervised. Interview participants indicated being satisfied with how the Taipei government equipped green spaces, including rehabilitation equipment, to support health-promoting activities.
(4) *Promote the health and social advantages of green spaces to older adults*. Taipei has the advantage of an established culture that supports and promotes the use of green spaces by older adults. That usage is maintained by high residential density, by a tradition of organized classes in martial arts and dancing, and by family networks that promote the care of children by grandparents. In addition, the historical and religious links between Confucian ideas of discipline and duty may extend to a belief that a disciplined body is an important personal responsibility. This may in turn confer a strong advantage in promoting the use of green spaces in Taipei over non-Asian cities. In absence of these more traditional and cultural supports, a more concerted and intensive health promotion effort may be required to establish a critical mass of activities and participants within green spaces that are sustainable. In places where green space usability varies with the seasons, these health promotion efforts may also need to be seasonal, or may need to consider using a mix of outdoor and indoor spaces.

### 3.5. Strengths and Limitations

#### 3.5.1. Strengths

This exploratory research provided an opportunity to observe the unique culture surrounding older adults’ use of community green spaces in Taipei, Taiwan. Both qualitative and quantitative methodologies were used to understand how green spaces can support the health and wellbeing of older adults. While the interviews allowed researchers to explore why older adults’ use the green spaces, the SOPARC tool allowed for observational information to be collected on physical activity in these green spaces. Interviews and SOPARC observations were conducted in a variety of green spaces located in different socioeconomic areas of the city.

#### 3.5.2. Limitations

Because the study capitalized on a time-limited visit to Taipei, observers had limited training with the SOPARC tool. Unfortunately, the study scope did not allow the observers to obtain sufficient information for statistical comparisons between the different times of day and different days of the week for each green space. As well, interview questions were highly structured and typically elicited brief responses. Language differences and the use of a translator limited detailed explorations of perceptions.

Another major limitation in this study is that observers were unable to conduct comprehensive interviews with more activity instructors and were unable to interview municipal officials. This would have provided information about the level of planning and support for green space development, supervision, and planning, as well as the extent to which health promotion activities are important in sustaining high participation rates by older adults. These questions would be a natural focus for further research.

## 4. Conclusions

This exploratory study provides a platform for future research to further explore perceptions and usage of green spaces in Taipei and other places throughout the world. Despite varying cultural contexts and geographic locations, there are many things that other places can learn from the unique culture surrounding older adults’ high use of community green spaces in Taipei, Taiwan. In particular, in Taipei the combination of structured and unstructured activities in the green spaces helped to foster positive social relationships as well as physical activity participation. In addition, the close proximity of the green spaces to older adult residences’ facilitated older adults’ use of the spaces.

Creating age-friendly spaces starts with addressing the health-related needs of older adults with urban planners and architects [44]. The green spaces in Taipei are a prime example of how environments can be designed to accommodate the unique needs of the aging population. These green spaces provide older adults with a sense of safety, foster social and community ties, offer health-promoting activities that promote physical health and mental wellbeing, and help older adults to ‘age in place’ and continue to live independently in their community.

## Supplementary Material

A short documentary containing video footage from this research project can be viewed at: https://www.youtube.com/watch?v=mw2bO6GfPpE.
